# Functional Analysis of Chicken IRF7 in Response to dsRNA Analog Poly(I:C) by Integrating Overexpression and Knockdown

**DOI:** 10.1371/journal.pone.0133450

**Published:** 2015-07-17

**Authors:** Tae Hyun Kim, Huaijun Zhou

**Affiliations:** Integrative Genetics and Genomics Graduate Group, Department of Animal Science, University of California, Davis, California, United States of America; University of Washington, UNITED STATES

## Abstract

In order to develop novel strategies to protect against increasingly virulent bird-linked pathogens, a better understanding of the avian antiviral response mechanism is essential. Type I interferons (IFNs) are recognized as the first line of defense in a host’s antiviral response; and it has been suggested that IRF7, a member of the IFN regulatory factor (IRF) family, plays an important role in modulating the immune response to avian influenza virus infection in chickens. The objective of this study was to identify candidate genes and pathways associated with IRF7 regulation at the transcriptome level as a first step towards elucidating the underlying cellular mechanisms of IRF7 modulation in the chicken antiviral response. *IRF7* overexpression and knockdown DF-1 cell lines were established and stimulated by various pathogen-associated molecular patterns. Significant *IRF7* and type I IFN expression changes were observed in both the *IRF7* overexpression cell line and the *IRF7 *knockdown cell line upon exposure to the double stranded RNA (dsRNA) analog poly(I:C). Using RNA-seq based transcriptome analysis, we identified potential novel genes that IRF7 may help regulate as part of the host immune response to dsRNA; potential biomarkers and therapeutic targets revealed as a result of this study warrant further investigation. Based on our results, we suggest that IRF7 may have conserved functional activity in the avian antiviral response, and plays a crucial role in type I IFN regulation.

## Introduction

Zoonotic viral pathogens are a serious threat to both poultry production and to human health. Avian influenza viruses (AIV) are not only a significant factor in the mortality of commercial chickens and some strains have been transmitted to humans and caused significant human death [1]. Historically, the reduced repertoire of immune-related genes in birds and the overall low sequence similarity to their mammalian orthologues has proved a significant challenge in elucidating molecular mechanisms of the antiviral response in chickens [2, 3]; it is urgent that we gain a better understanding of this response in order to facilitate the development of therapeutics for the control of AIV in chickens and to advance strategies for the limiting of and response to zoonotic AIV transmission to humans.

Upon activation by pathogen recognition receptors (PRRs), type I interferons (IFNs) serve as the primary trigger of a host’s innate immune response against viral infection by initiating a signaling pathway that includes more than 300 IFN-stimulated genes (ISGs) in mammals [4]. Interferon regulatory factors (IRFs), transcription factors of type I IFNs, further modulate the immune response and are especially important for broad aspects of host defense such as adaptive immunity, oncogenesis regulation, and cell lineage differentiation [5]. IRF3 and IRF7, two key transcription factors that modulate type I IFN expression, are phosphorylated, undergo homo/heterodimerization, and translocate into the nucleus upon viral infection [6]. It has been demonstrated in mice that upon ssRNA viral infection, along with IRF3, IRF7 plays a key role in the induction and modulation of type I IFN expression [7]. Robust expression of type I IFNs is ensured via positive feedback regulation, as downstream components of the IFN signaling cascade drive the expression of IRF7 [6].

As in mammalian hosts, chickens exhibit a robust induction of type I IFNs in response to infection by a variety of different viruses, such as either Newcastle disease virus (NDV) or AIV [8]. To date, eight IRF homologues have been identified in chickens, though their functions are not yet well defined [9]. Chicken IRF7 was first identified in 1995, prior to the discovery of its human orthologue, but was originally labeled IRF3 due to its high degree of similarity to mammalian IRF3 [10]; it was relabeled as IRF7 when subsequent studies revealed that IRF3 is absent in chickens and other avian species [11]. IRF9, another member of the mammalian IRF family thought to be involved in signal transduction, activation, and signaling in the positive feedback loop of the IFN cascade, also appears to be absent in chickens [9].

Previous work from our lab has shown that IRF7 is involved in the regulation of the host response to AIV infection in broiler chickens [12]. In a recent *in vitro* study, researchers demonstrated that IRF7 expression in the immortalized chicken embryonic fibroblast DF-1 cell line (DF-1) was highly up-regulated in response to low pathogenic AIV (H9N2) infection whereas IRF7 was less responsive against the high pathogenic AIV (H5N1) infection [13]. Variation in IRF7 expression upon exposure to AIV serotypes of differing pathogenicity suggests that IRF7 is an important regulator of the antiviral innate immune response in chickens, and that it could help determine a chicken’s susceptibility to different AIV strains.

The advent of high-throughput mRNA sequencing (RNA-seq) has greatly facilitated our ability to investigate transcriptional responses more accurately, dynamically, and in a more context–specific manner [14]. The objective of this study was to identify novel genes and pathways associated with IRF7 and its response to the double stranded RNA (dsRNA) analog poly(I:C) in chickens. To do so, we modified DF-1 cells using the piggyBac transposon system to establish both IRF7 overexpression and IRF7 knockdown lines; these lines were then exposed to various pathogen associated molecular patterns (PAMPs), and their responses characterized. RNA-seq analysis was performed to identify novel transcripts, functional gene ontologies, and signaling pathways modulated by IRF7.

## Materials and Methods

### Total RNA isolation

Bursa and spleen tissues obtained from 1-day old broilers in a previous study [12] were stored in RNA*later* solution (Ambion, Austin, TX) at -80°C and used for RNA isolation. Total RNA was isolated from tissues using Trizol (Invitrogen, Carlsbad, CA) according to the manufacturer’s protocol. Direct-zol RNA MiniPrep Kit (Zymo research, Irvine, CA) was used to isolate RNA from cell lines. The RNA quality was checked by agarose gel electrophoresis, and the RNA concentration and purity were determined by NanoDrop 2000c (Thermo scientific, Waltham, MA).

### Cloning chicken IRF7

Complement DNA (cDNA) was synthesized from total RNA (500 ng) using the Superscript III first-strand synthesis system (Invitrogen). The coding sequence (CDS) of chicken IRF7 was amplified from the bursa and spleen tissue-derived cDNAs using primers designed based on the provisional mRNA sequence (NM_205372.1) (Table 1). PCR products were cloned into the pGEM-T Easy Vector (Promega, Madison, WI) for sequencing.

**Table 1:** List of gene specific PCR primer sequences for cloning and quantitative reverse transcriptase PCR.

Primer set	Gene Accession	Forward	Reverse
Cloning			
* IRF7* CDS	NM_205372	ATGGCAGCACTGGACAGCGA	TCAGTCTGTCTGCATGTGGT
qRT-PCR			
* CLDN5*	NM_204201	CTGGTGGCGCTCATGGTCAC	CGATGTTGGCGAACCAGCAG
* DCLRE1C*	NM_001031594	GCGAGTACCCGCAGCTGTCC	CAAGCTGCTCTGCAGCCTCC
* EIF2AK2*	NM_204487	CGTCGACGTGGACATGAGAGG	TGTCCCACGTTTTTGCTGCTG
* GAPDH*	NM_204305	CTGGGGCTGCTAAGGCTGTG	CACCCGCATCAAAGGTGGAG
* G0S2*	NM_001190924	AAAGAGCTGAGCGCCGCAAC	TTCCTGTTGGGCTTCTGGCTG
* IFNA3 (IFN-alpha)*	NM_205427	AAATCCTCAGCAGCCCCAGC	GTGCAGGAACCAGGCACGAG
* IFNB (IFN-beta)*	NM_001024836	GCCTTGCCCACAACAAGACG	GCGTGTGCGGTCAATCCAGT
* IRF7*	NM_205372	CGACCCGCACAAGGTCTACG	GGAGCCGAGGGCAGAGATGT
* ITGA8*	NM_205288	TGTGGGTGCGTTTGGAGCTG	ACAGGCCACGAAAAGCGGAG
* LY6E*	NM_204775	GCATCCTCCAACTGGGCCTG	AATCCCAGCACTGGGGCAAA
* MX1*	NM_204609	GGAATTGCCAGAGAGGCCGT	CTTGAGCCATTTTCAGCGCC
* WIF1*	NM_001199607	TGGCTGCGGACTGTATGGGA	CTTCTTGGGCGAAGGCGTGT
* TLR3*	NM_001011691	TCAGTACATTTGTAACACCCCGCC	GGCGTCATAATCAAACACTCC
* IRF7* 3'UTR	NM_205372	GGGCCATACTGACCAGCCCA	TGTCCTGGGAGCGAAGGAGG
Rabbit *HBB2* pA	NM_001082260	CGGAAGGACATATGGGAGGGC	ATGGACAGCAGGGGGCTGTT

### Cell culture and siRNA transfection

Immortalized chicken embryonic fibroblast DF-1 cells (ATCC, Teddington, UK) were cultured in Dulbecco's Modified Eagle's Medium (Thermo Scientific) supplemented with 10% fetal bovine serum (Atlanta Biologicals, Flowery Branch, GA), 100U/ml penicillin, 100μg/ml streptomycin (Thermo Scientific), and incubated at 37°C in a humidified atmosphere containing 5% CO_2_. Three small interfering RNAs (siRNAs) targeting *IRF7* CDS and one non-specific (NS) control were designed using siDESIGN Center (GE Dharmacon, Lafayette, CO) (Table 2). 10pmol of each siRNA were transfected into 1x10^5^ DF-1 cells using Lipofectamine RNAiMAX reagent (Invitrogen) following the manufacturer’s protocol; transfected cells were harvested for RNA extraction 48h after transfection. Three biological replicates were used in each transfection.

**Table 2:** Information of IRF7 knockdown siRNA.

Candidate siRNA	Direction	Sequence
IRF7-497	Sense:	AGACAGUACUGAAGGUGUUUU
	Antisense:	AACACCUUCAGUACUGUCUUU
IRF7-1246	Sense:	GCACAGAGCUCCGGGACUUUU
	Antisense:	AAGUCCCGGAGCUCUGUGCUU
IRF7-1333	Sense:	GCACAAAGCCCAAGGAGUCUU
	Antisense:	GACUCCUUGGGCUUUGUGCUU
Non-specific (NS) control	Sense:	UGCUUUAACCACCGCAUCCUU
	Antisense:	GGAUGCGGUGGUUAAAGCAUU

### Construction of expression vectors

The piggyBac (PB) transposon expression vector (PB513B) and PB transposase plasmid (System Biosciences, Mountain View, CA) were used for stable expression of transcripts. For the overexpression vector, the *IRF7* CDS was cloned into PB513B under the expression of the cytomegalovirus immediate-early enhancer/promoter (CMV promoter). The most efficient siRNAs targeting *IRF7* and the NS control siRNA were converted into their corresponding short-hairpin RNA (shRNA). The CMV promoter was removed from PB513B and replaced with the chicken U6-3 promoter to drive shRNA expression in the knockdown vector [15].

### Transfection and Puromycin selection

Each PB transposon expression vector was co-transfected with PB transposase plasmid into DF-1 cell lines using the Lipofectamine 2000 reagent (Invitrogen) according to the manufacturer’s protocol. Puromycin (3μg/ml) was added to the culture media 48h after transfection and *IRF7* overexpression cell lines with stable GFP-expression were selected at 2wk post transfection.

### Quantitative reverse transcriptase PCR

Quantitative reverse transcriptase PCR (qRT-PCR) was performed using the Applied Biosystems 7500 Fast Real-Time PCR System (Life Technologies, Grand Island, NY) with SYBR Select Master Mix (Life Technologies). cDNAs from each sample were amplified with gene specific primers (Table 1); each reaction was run in triplicate at a final volume of 20μL per reaction with 2μL of 10 fold diluted cDNA, 100nM of each primer, and 10μL of SYBR Select Master Mix. PCR cycling conditions were as follows: 50°C, 2 min; 95°C, 2 min; 40 cycles of 95°C, 15 sec and 60°C, 1 min; followed by a melting curve program. Chicken glyceraldehyde 3-phosphaste dehydrogenase (*GAPDH*) was used as a reference control for each reaction.

### PAMPs stimulation of cell lines

The stable *IRF7* overexpression and knockdown DF-1 cell lines and their controls were exposed to 1μg/mL PGN-SA (Invivogen, San Diego, CA), 1μg/mL poly(I:C) (Invivogen), 1μg/mL LPS-SM (Invivogen), 1mM Loxoribine (Invivogen), or media (mock) as a control; cells were collected at 6h and 24h post-stimulation for RNA extraction. Three biological replicates were used in each group.

### cDNA library preparation and RNA-seq

A total of eight libraries (one library per poly(I:C) or mock treatment group) were constructed from 6h post stimulation cells: control-mocked, control-stimulated, overexpression-mocked, and overexpression-stimulated (overexpression groups); and NS-mocked, NS-stimulated, knockdown-mocked, and knockdown-stimulated (knockdown groups). Each library was generated from pools of RNA representing three biological replicates; the total RNA (1μg) for each pooled sample served as the starting material to prepare a cDNA library using the NEBNext Ultra Directional RNA Library Prep Kit (New England Biolabs, Ipswich, MA). Illumina HiSeq 2000 was used to generate 100bp paired-end reads.

### Mapping reads and transcriptome analysis

The raw sequence data were subjected to quality control using the FASTQC and the Tuxedo tools were applied for transcriptome analysis [14, 16]. Paired-end raw reads were aligned against the galGal4 reference chicken genome, Ensembl gene model (version 75), by Tophat2 (Version 2.1.0) [17]. Trinity software modules were used to generate a de novo assembly from the RNA-seq reads of this study as well as from the RNA-seq reads from our previous study [18, 19]. Transcript assembly and differential gene expression analysis were performed via the Cufflinks program (Version 2.1.0) [20]. The blind dispersion method was used to identify differentially expressed genes (q-value < 0.05) [21]. Functional annotations for significantly differentially expressed genes were performed using DAVID [22, 23]. The enriched gene ontology (GO) terms on biological processes and the pathways obtained from DAVID functional analysis were filtered for significance by gene count ≥ 3, p-value < 0.05, and FDR <20%.

## Results

### Molecular cloning of *IRF7* coding sequence in chickens

**Fig 1:**
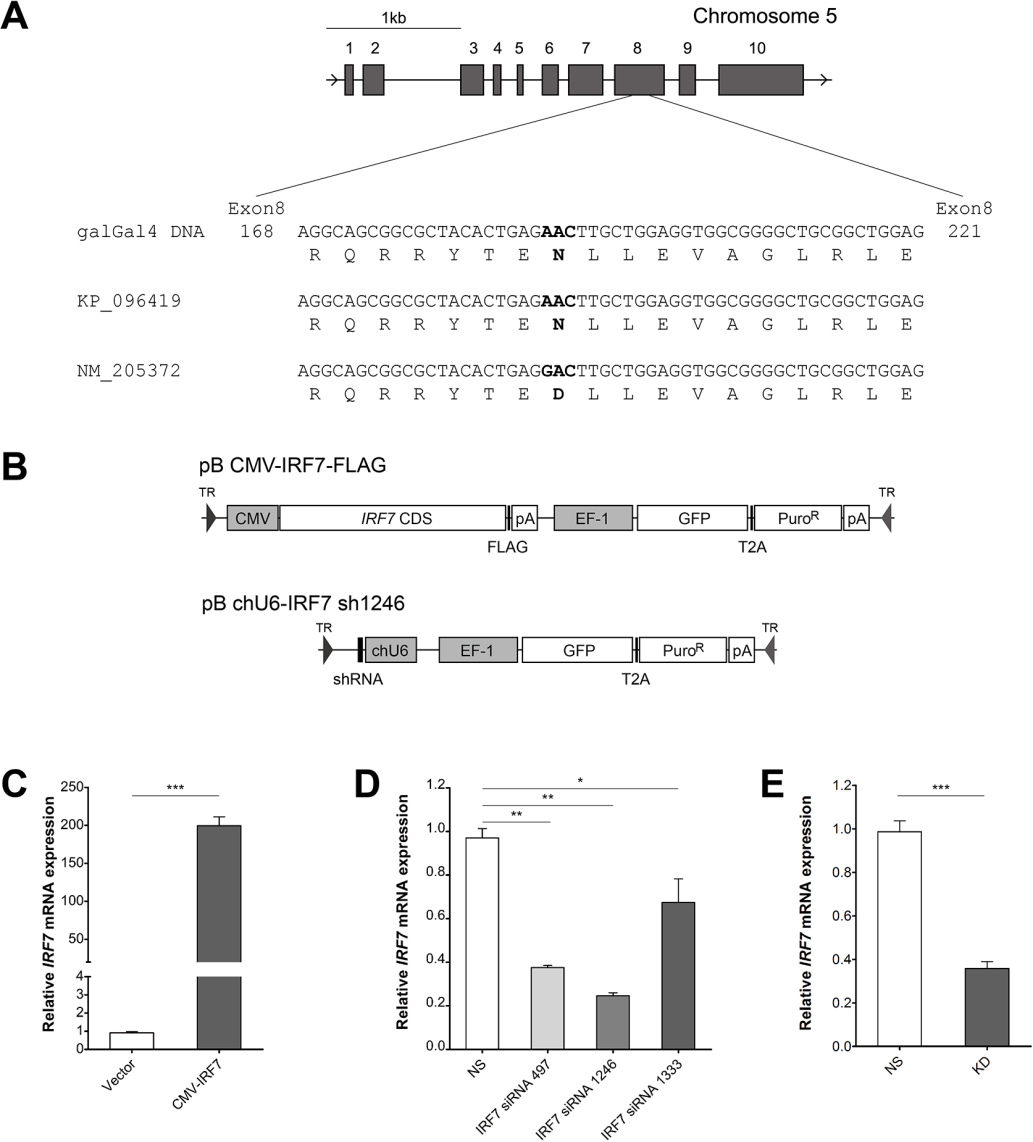
Establishment of *IRF7* overexpression and knockdown DF-1 cell lines. (A) Multiple sequence alignment of IRF7 sequences in exon 8 nucleotide position 169 to 221. Molecular cloned IRF7 clone (accession) and the NCBI reference sequence (NM_205372) were aligned against the reference chicken genome (galGal4). (B) Schematic diagram of overexpression (up) and knockdown (down) PiggyBac transposon expression vectors. (C) Related quantitation of IRF7 in control (Vector) and overexpression (CMV-IRF7) cell lines. (D) Knockdown efficiency of each siRNA for IRF7 in the overexpressed cell line. siRNA non-specific (NS) to the chicken genome was used as a control. (E) Knockdown efficiency of IRF7 in the knockdown cell line (KD) as compared to control cells (NS). * *P* < 0.05, ** *P* < 0.01, *** *P* <0.001. Error bars indicate the SEM of triplicate analyses.

The cloned CDS of chicken *IRF7* mRNA was submitted to NCBI (KP_096419) and subjected to a BLAT search against the reference chicken genome from the UCSC Genome browser (http://genome.ucsc.edu) [24]. Chicken *IRF7*, located on chromosome 5, has a CDS length of 1,476 base pairs encoding 491 amino acids. Sequence alignment showed a discrepancy between the NCBI reference sequence and both the galGal4 and our cloned CDS at the 343rd amino acid position (the NCBI reference sequence (NM_205372) lists the 1027th nucleotide position of the CDS as guanine while the galGal4 reference and our cloned CDS indicate an adenine at that position) (Fig 1A). Given that the galGal4 reference genome sequence and our cloned sequence matched, the cloned CDS was used for expressing *IRF7* in the DF-1 cell line.

### Overexpression and knockdown of IRF7 in the DF-1 cell line

In order to study the function of IRF7 *in vitro*, stable *IRF7* overexpression and knockdown DF-1 cell lines were established; diagrams of the vector constructs used are shown in Fig 1B. In the overexpression construct, *IRF7* expression is driven by the CMV immediate-early enhancer/promoter, with GFP and Puromycin resistance serving as dual selection markers; empty vector, encoding only the selection markers, was used to generate control cell lines. Following transfection and culturing, a single *IRF7* overexpression DF-1 cell line was selected based on its stable expression of both the selection markers and IRF7; the cell line selected demonstrated approximately 200-fold higher expression of *IRF7* relative to the control cell line as determined by qRT-PCR (Fig 1C).

To select the most effective construct for the knockdown of *IRF7*, three siRNAs targeting *IRF7* and one non-specific (NS) control siRNA were designed and transfected into the DF-1 cell line. Knockdown efficiency was measured 48h after siRNA transfection by qRT-PCR. Of the three siRNA candidates, IRF7 siRNA 1246 demonstrated the highest knockdown efficiency (a 75% reduction relative to the control) (Fig 1D). To establish stable knockdown cell lines, the siRNA 1246 and NS control siRNA sequences were converted to shRNA constructs driven by the chicken U6-3 promoter (DQ531569) and placed in the PB vector carrying dual selection markers as in the overexpression construct (Fig 1B). As before, transformed cells were screened for stable expression of the construct, and a single lineage selected; the established *IRF7* knockdown DF-1 lineage exhibited approximately 70% efficiency compared to the NS control (Fig 1E).

### PAMPs stimulation on stable cell lines

**Fig 2:**
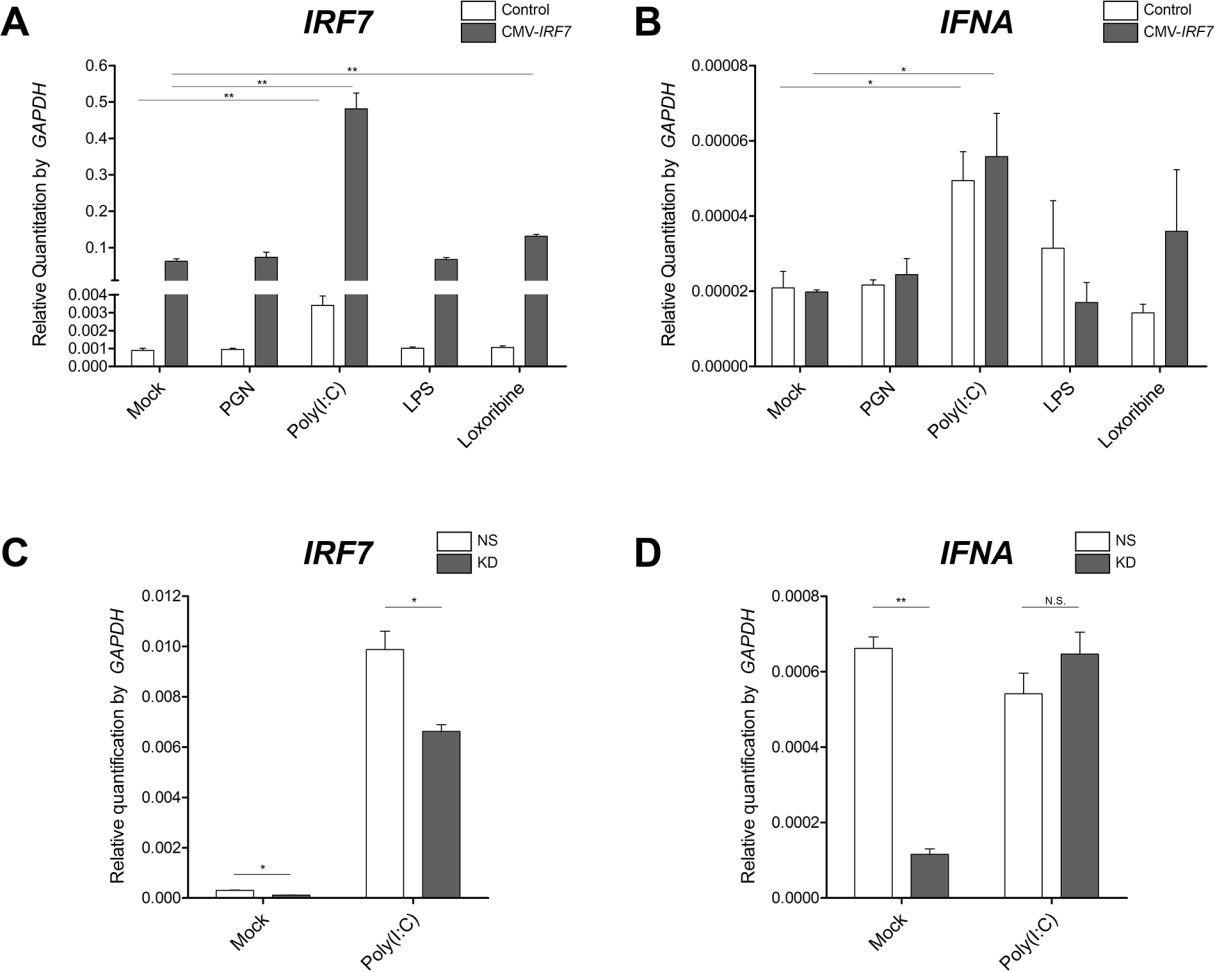
Expression changes of *IRF7* and *IFNA* in response to different pathogen associated molecular patterns (PAMPs) stimulated cell lines. (A, B) *IRF7* overexpression (CMV-IRF7) or (C, D) knockdown (KD) DF-1 cell lines and their respective control lines at 6h post stimulation by PAMPs. Mock: media (negative control); PGN: peptidoglycan; poly(I:C): polyinosinic-polycytidylic acid; LPS: lipopolysaccharide. * *P* < 0.05, ** *P* < 0.01. Error bars indicate the SEM of triplicate analyses.

The established cell lines were exposed to various PAMPs in order to examine which pathway(s) IRF7 is involved in. The overexpression cell lines were exposed to PGN, poly(I:C), LPS or Loxoribine. At 6h post-stimulation, mRNA expression of both *IRF7* and IFN-alpha (*IFNA*) was significantly up-regulated in response to poly(I:C) stimulation in both the control and overexpression cell lines as compared to mock treated cell (Fig 2A and 2B). Loxoribine also induced *IRF7* expression, but showed no significant difference in *IFNA* expression in the overexpression group as compared to the mock group. Similar trends were observed at 24h post-stimulation (data not shown). Because only poly(I:C) stimulation exhibited a statistically significant effect on both *IRF7* and *IFNA* expression in both the control and overexpression cell lines, the knockdown cell lines were only treated with poly(I:C). A significant decrease in *IRF7* expression was observed in poly(I:C) treated *IRF7* knockdown cells as compared to NS control cells (Fig 2C). *IFNA* expression was significantly decreased in mock-treated knockdown cells relative to the NS line, but no significant difference in expression was observed between knockdown versus NS cells treated with poly(I:C) (Fig 2D).

### RNA-seq analysis in poly(I:C) stimulated cell lines

A total of eight cDNA libraries were prepared from poly(I:C)-stimulated and mocked-treated cells from the *IRF7* overexpression and knockdown cell lines as well as their respective control lines. Each library was constructed from RNA pooled from three biological replicates. Paired-end 100bp reads were generated by the Illumina Hiseq2000; 28.3±1.5 million (M) and 15.0±1.4 M reads were produced from the overexpression library and knockdown library respectively. An average of 74% of the reads were mapped to the galGal4 chicken genome with a 68% concordant pair alignment rate by Tophat2 [17]. The data files from RNA-seq analysis have been deposited in NCBI's Gene Expression Omnibus [25], and are accessible through GEO Series accession number GSE70105 (http://www.ncbi.nlm.nih.gov/geo/query/acc.cgi?acc=GSE70105).

### Novel transcript discovery

Similar to IRF7, IRF3 is very important in regulating the host immune response to virus infection in mammals [6]. An *IRF3* gene has not been annotated in chickens; whether this is because there is no chicken IRF3 orthologue, or because limited data availability has prevented its identification in the current genome release (Galgal4) remains uncertain. To address the later possibility, RNA-seq data from this study and RNA data from AIV infected lung tissues from our group were used for de novo RNA-seq assembly using Trinity software [18, 19]. The de novo RNA-seq assembly output was used as a database and subjected to BLAST search [26]; both the whole protein sequence and the conserved IRF-3 domain of human, mouse, and zebra fish *IRF3* were queried against the generated transcriptome assembly and no novel IRF candidate genes were identified in either transcriptome. The Cufflinks program was also utilized to try and identify any novel IRFs or spliced isoforms of *IRF7* from the aligned reads; only previously annotated members of the IRF family, including a single *IRF7* isoform, were found in either transcriptome.

### Transcriptome analysis of differentially expressed genes in the cell lines

**Fig 3:**
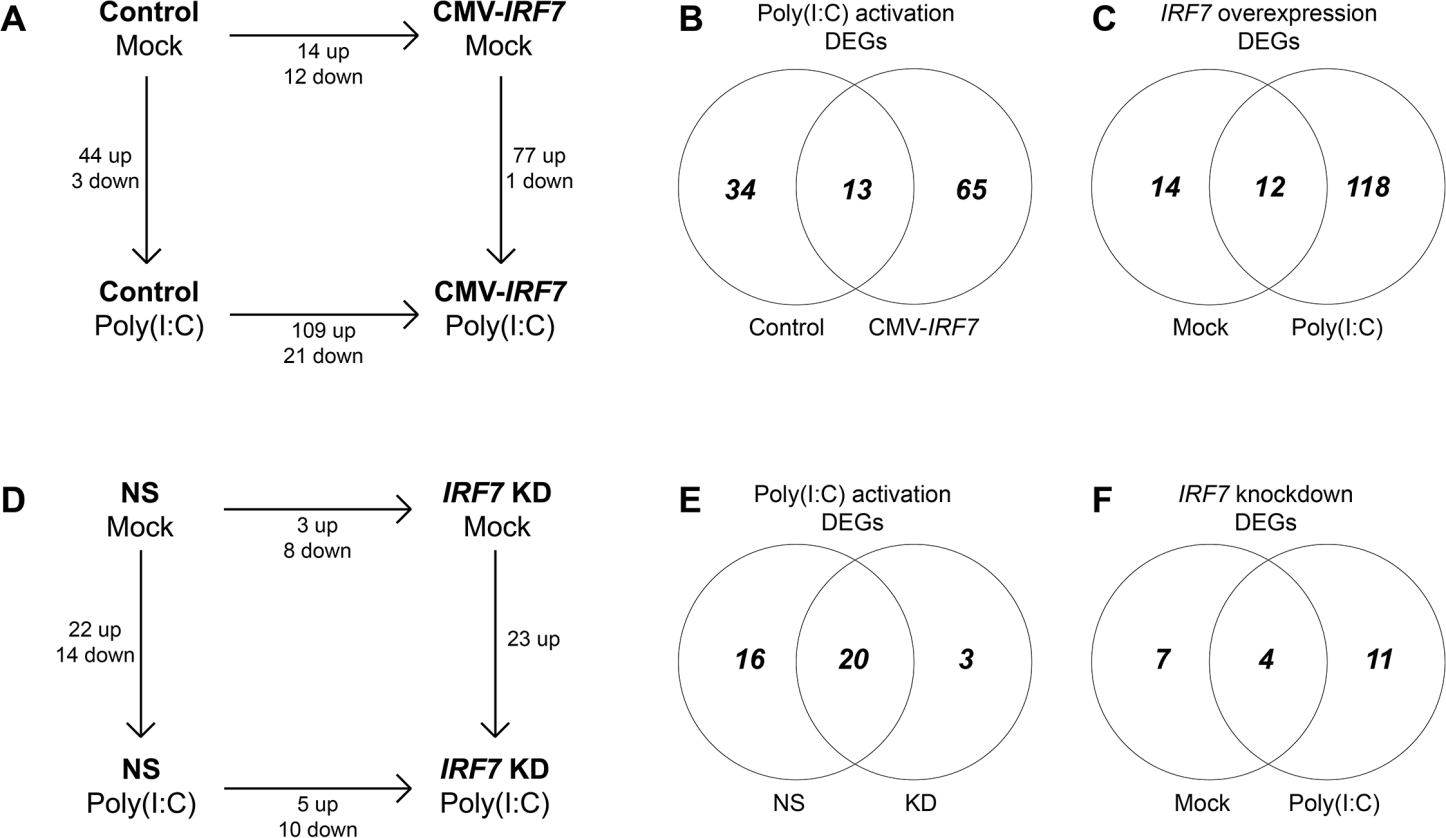
Differential expression analyses of RNA-seq samples. Differentially expressed genes (DEGs) between cell lines and treatments in the (A) overexpression and (D) knockdown data set. (B, E) Venn diagrams of overlapped DEGs between cell lines upon poly(I:C) expression. (C, F) Venn diagrams of overlapped DEGs between treatments in effect of IRF7 expression changes.

Differential expression analysis was performed on the aligned reads using Cufflinks [20]. Differentially expressed genes (DEGs) were identified from four pairwise comparisons under either overexpression or knockdown conditions (Fig 3A and 3D; S1 and S2 Tables); the magnitude of change for the majority of the DEGs identified was greater than 4-fold (in either direction), with the lowest magnitude of change being 2.18.

Our results indicate that in the *IRF7* overexpression cell lines, 77 genes were up-regulated and 1 gene was down-regulated in response to poly(I:C) stimulation; in the control cell line, 44 genes were up-regulated and 3 genes were down-regulated (Fig 3A). Of the 112 genes that were differentially expressed in either the control or *IRF7* overexpression line upon treatment with poly(I:C), 65 were specific to the overexpression cell line, 34 were specific to the control cell line, and 13 were differentially expressed to both lines (Fig 3B). The overexpression of *IRF7* resulted in the up-regulation of 14 genes and down-regulation of 12 genes in the mock treatment and the up-regulation of 109 genes and down-regulation of 21 genes in the poly(I:C) treatment. Of the 144 genes differentially expressed in CMV-*IRF7* cells versus control cells, 118 were specific to cells treated with poly(I:C), 14 were specific to cells under mock treatment conditions, and 12 exhibited differential expression under both mock and poly(I:C) treatment conditions (Fig 3C). ISGs such as 2'-5'-oligoadenylate synthetase-like (*OASL*) and eukaryotic translation initiation factor 2-alpha kinase 2 (*EIF2AK2*), as well as genes involved in immune signal transduction such as nuclear factor of kappa light polypeptide gene enhancer in B-cells inhibitor, zeta (*NFKBIZ*) and tripartite motif containing 25 (*TRIM25*), overlapped in the both DEG lists (Fig 3B and 3C, S1 Table).

For the knockdown condition, following poly(I:C) stimulation, there were 22 up-regulated and 14 down-regulated DEGs in the NS control cell line, and 23 up-regulated DEGs in the knockdown cell line; of the 39 genes differentially expressed in response to poly(I:C) treatment, DEGs, 3 and 16 were specific to the knockdown cell line and the NS line respectively (Fig 3E, S2 Table). For *IRF7* knockdown line relative to the NS control line, there were 3 up-regulated and 8 down-regulated DEGs in mock treated cells, and 5 up-regulated and 10 down-regulated DEGs undert the poly(I:C) treatment (Fig 3D). Of the 22 genes expressed differentially in the knockdown line relative to the NS control line, 11 and 7 were specific to the poly(I:C) and the mock treatment groups respectively (Fig 3F).

### Validation of RNA-seq by qRT-PCR

**Fig 4:**
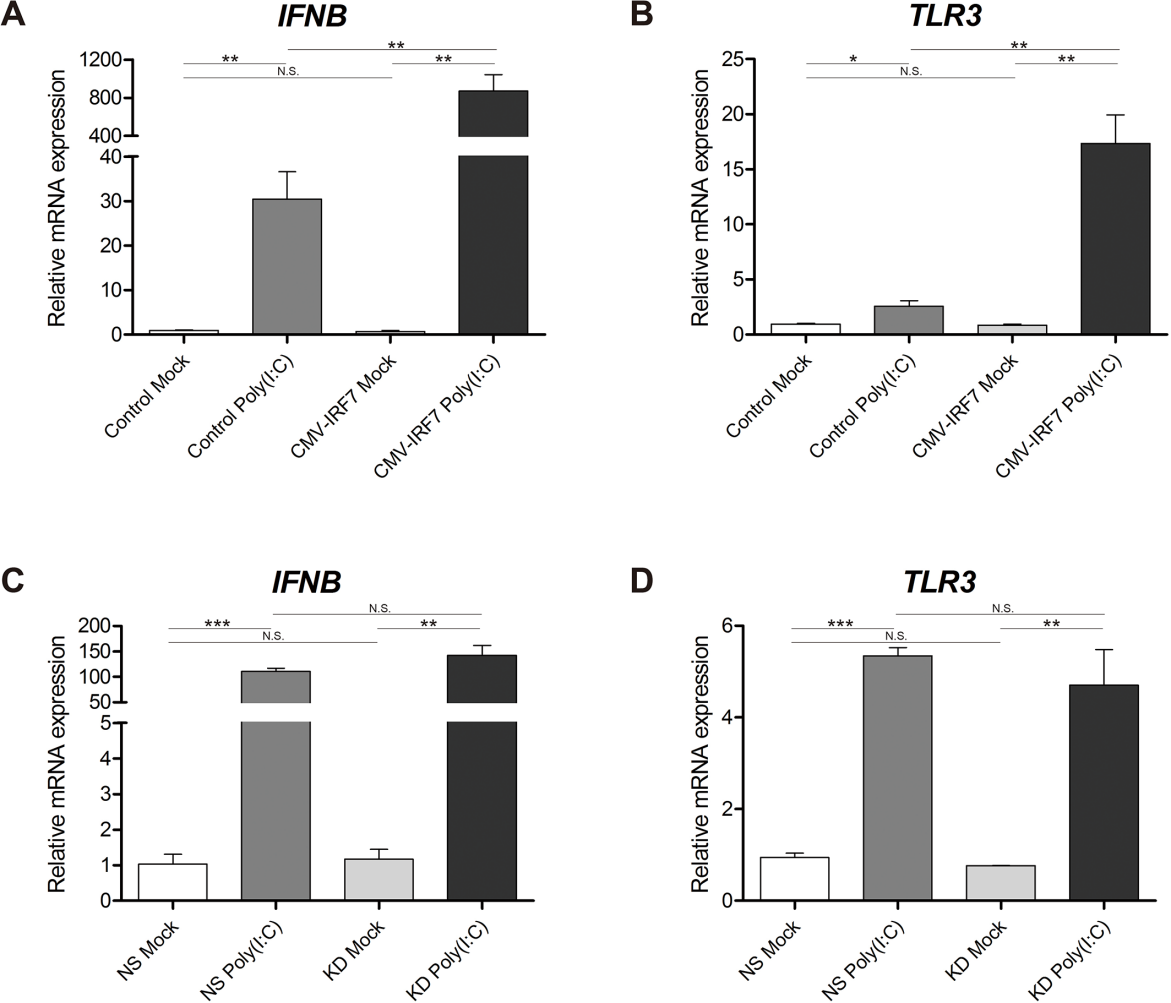
Expression of *IFNB* and *TLR3* in the overexpression and knockdown cell lines. Relative expression levels of *IFNB* (A, C) and *TLR3* (B, D) were confirmed by qRT-PCR in the mock or poly(I:C) induced overexpression or knockdown cell lines and their respective controls. * *P* < 0.05, ** *P* < 0.01, *** *P* <0.001, N.S.: not significant. Error bars indicate the SEM of triplicate analyses.

To validate the results obtained by RNA-seq analysis, qRT-PCR was performed for each of the 8 pooled RNA libraries. Analysis by qRT-PCR confirmed that another type I IFN, IFN-beta (*IFNB*) expression was up-regulated in response to poly(I:C) treatment, but was not limited to the overexpression of *IRF7* itself; while the expression level of IFNB in response to poly(I:C) treatment was significantly higher in the *IRF7* overexpression line as compared to the control line, a significant increase in expression was also observed in the poly(I:C) treated control line relative to mock-treated control cells (Fig 4A). Toll-like receptor 3 (*TLR3*), recognized as a dsRNA recognition receptor [27], exhibited a similar expression trend as *IFNB* in the overexpression cell lines (Fig 4B), but not in the knockdown *IRF7* line, in which no significant change in expression level was observed for either *IFNB* or *TLR3* (Fig 4C and 4D).

In addition to *IFNB* and *TLR3*, eight genes were selected for testing by qRT-PCR to probe their transcriptional response to poly(I:C) treatment in the overexpression and knockdown cell lines. Three genes, *EIF2AK2*, lymphocyte antigen 6 complex locus E (*LY6E*), and myxovirus resistance 1 (*MX1*), were selected from the list of DEGs in poly(I:C) stimulated *IRF7* overexpression cells relative to control cells. To help identify potential false negatives in the RNA-seq analysis, five genes with non-significant differential expression were selected. These genes, WNT inhibitory factor 1 (*WIF1*), claudin 5 (*CLDN5*), integrin alpha 8 (*ITGA8*), G0/G1 switch 2 (*G0S2*), and DNA cross-link repair 1C (*DCLRE1C*), exhibited at least a 2 fold-change in expression in both the overexpression and knockdown lines relative to their respective control lines in our RNA-seq analysis, but did not meet the statistical threshold set (q-value >0.05).

**Fig 5:**
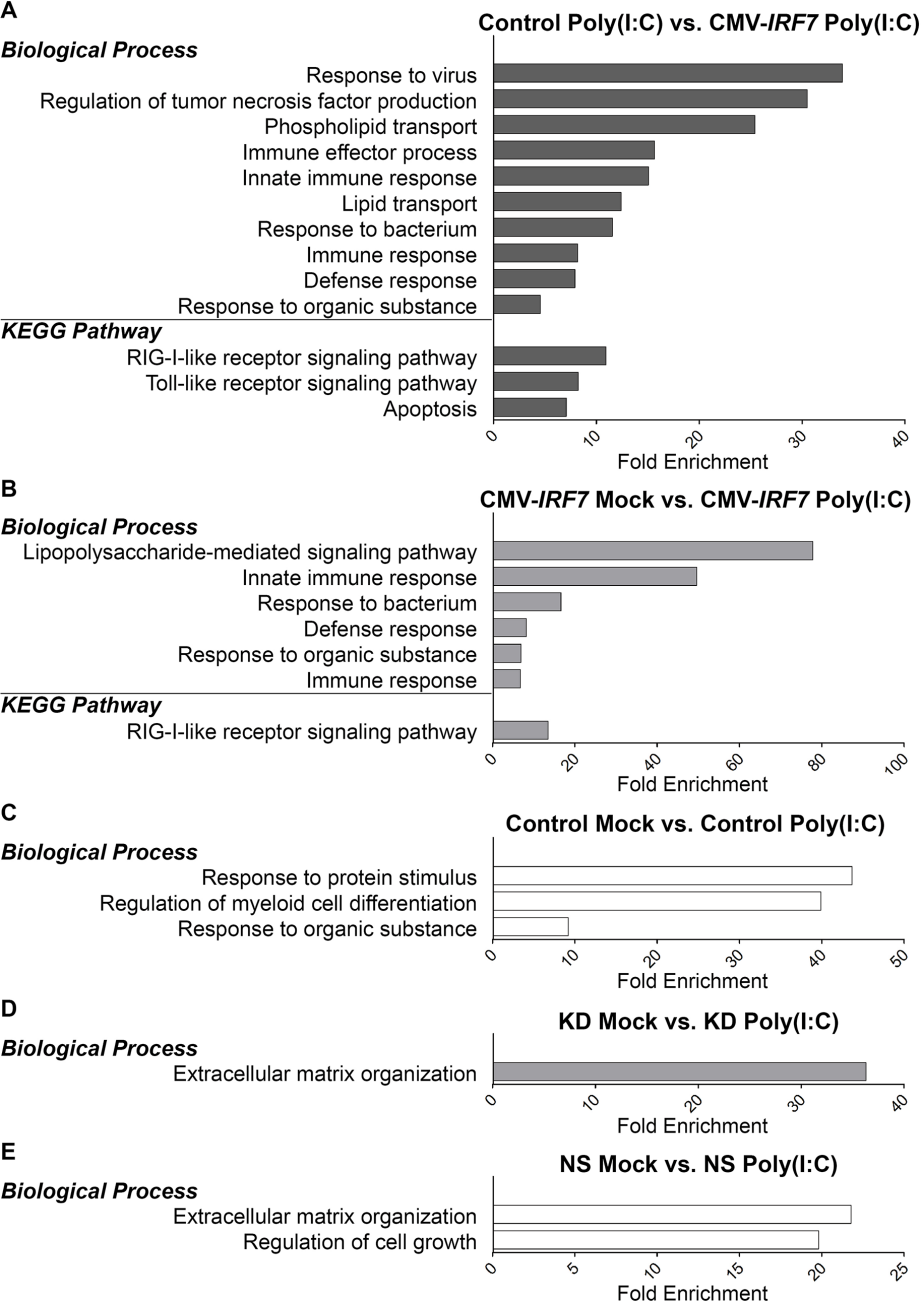
Validation of differentially expressed genes by quantitative reverse transcriptase PCR (qRT-PCR). Expression level of mRNAs were confirmed by qRT-PCR in the poly(I:C) induced overexpressed (left graph), knockdown (right graph) cell lines and their controls. (A) EIF2AK2; (B) LY6E; (C) MX1; (D) WIF1; (E) CLDN5; (F) ITGA8; (G) G0S2; (H) DCLRE1C. * *P* < 0.05, ** *P* < 0.01, *** *P* <0.001, N.S.: not significant. Error bars indicate the SEM of triplicate analyses.

All three of the statistically significant DEGs selected from our RNA-seq analysis were not only validated in the overexpression condition by qRT-PCR (each demonstrating at least a 7.5-fold change), but were also shown to exhibit significant differences in expression in the knockdown cells line even though the significances were not detected via RNA-seq (Fig 5A–5C). For the five genes deemed non-significant with regards to differential expression in our RNA-seq analysis, three demonstrated no significant differences via qRT-PCR either. In the case of *WIF1*, differences in expression were found for both the overexpression and knockdown lines, while in *CLDN5*, differences were found in just the overexpression line; while the changes observed for these genes were relatively low (roughly two-fold), they were statistically significant (Fig 5D–5H).

### Gene ontology (GO) analysis

**Fig 6:**
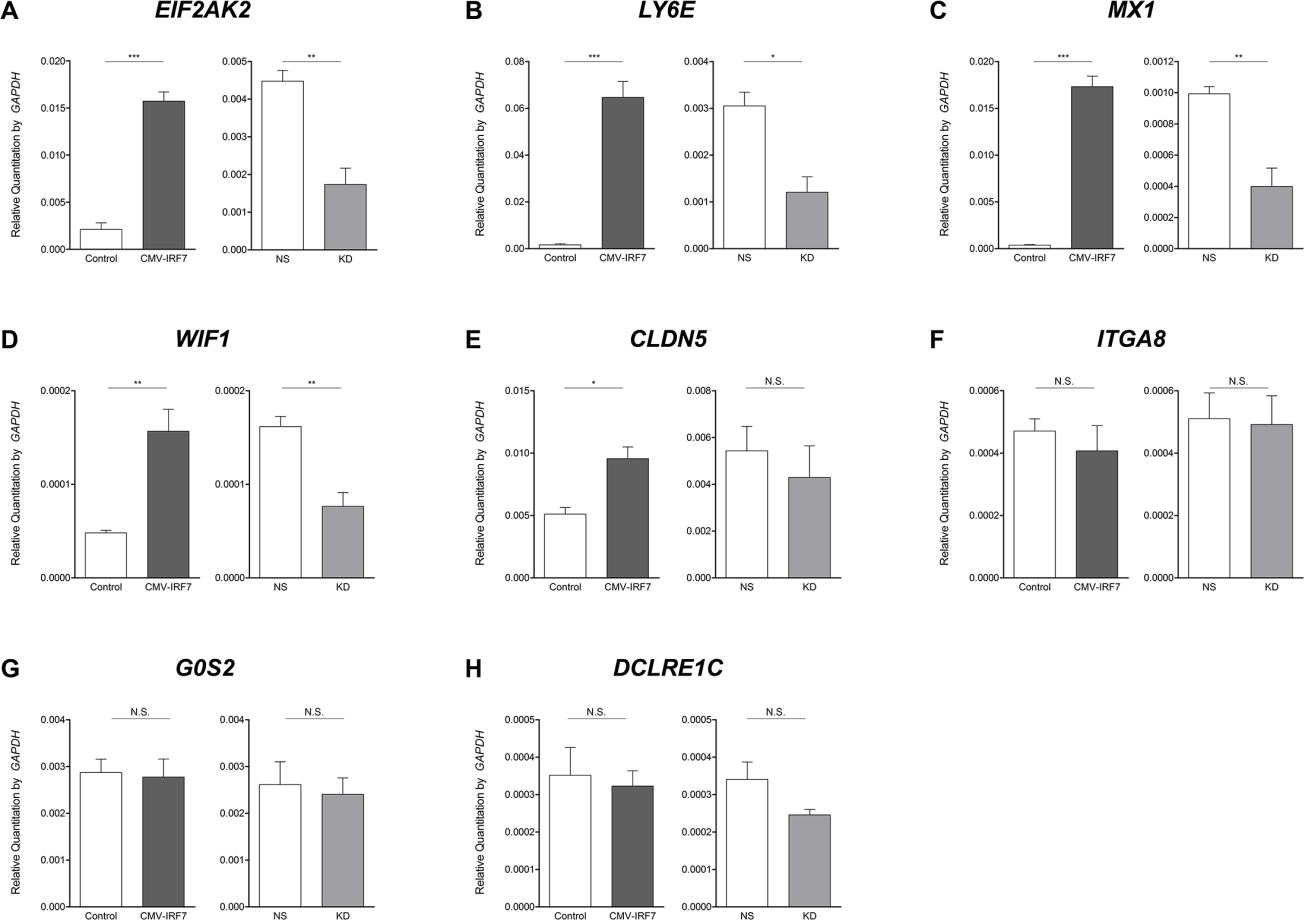
Gene ontology (GO) annotation terms on biological processes and pathways enriched by differentially expressed genes (DEGs). (A) GO terms enriched by DEGs between control and overexpressed cell lines upon poly(I:C) induction. (B) GO terms enriched by DEGs between mock and poly(I:C) treatments in the overexpressed cell lines. (C) GO terms enriched by DEGs between mock and poly(I:C) treatments in the control cell lines. (D) GO terms enriched by DEGs between mock and poly(I:C) treatments in the knockdown cell lines. (E) GO terms enriched by DEGs between mock and poly(I:C) treatments in the non-specific control cell lines. *P*<0.05.

The DEG lists obtained from both the overexpression and knockdown datasets were analyzed for enriched functional terms in biological processes and pathways using DAVID (http://david.abcc.ncifcrf.gov/tools.jsp) [22, 23].Under poly(I:C) stimulation, many biological processes, including ‘response to virus’, ‘immune effector process’, ‘innate immune response’, and ‘defense response’ were significantly enriched with *IRF7* overexpression. The nucleic acid sensing pattern-recognition receptors RIG-1-like receptor (RLR) and TLR pathways were also enriched under poly(I:C) stimulation in the *IRF7* overexpression line according to pathway analysis (Fig 6A). Under overexpression conditions, several immune-related GO terms and RLR pathway were significantly enriched upon poly(I:C) stimulation (Fig 6B); fewer GO terms were found to be enriched from the DEGs identified from control cell lines or knockdown cell lines (Fig 6C–6E). Differentially expressed genes that are in the RLR and TLR pathways were all upregulated under *IRF7* overexpression and poly(I:C) induction (S1 Fig).

### Identification of the origin of *IRF7* expressions in the overexpression cell line

**Fig 7:**
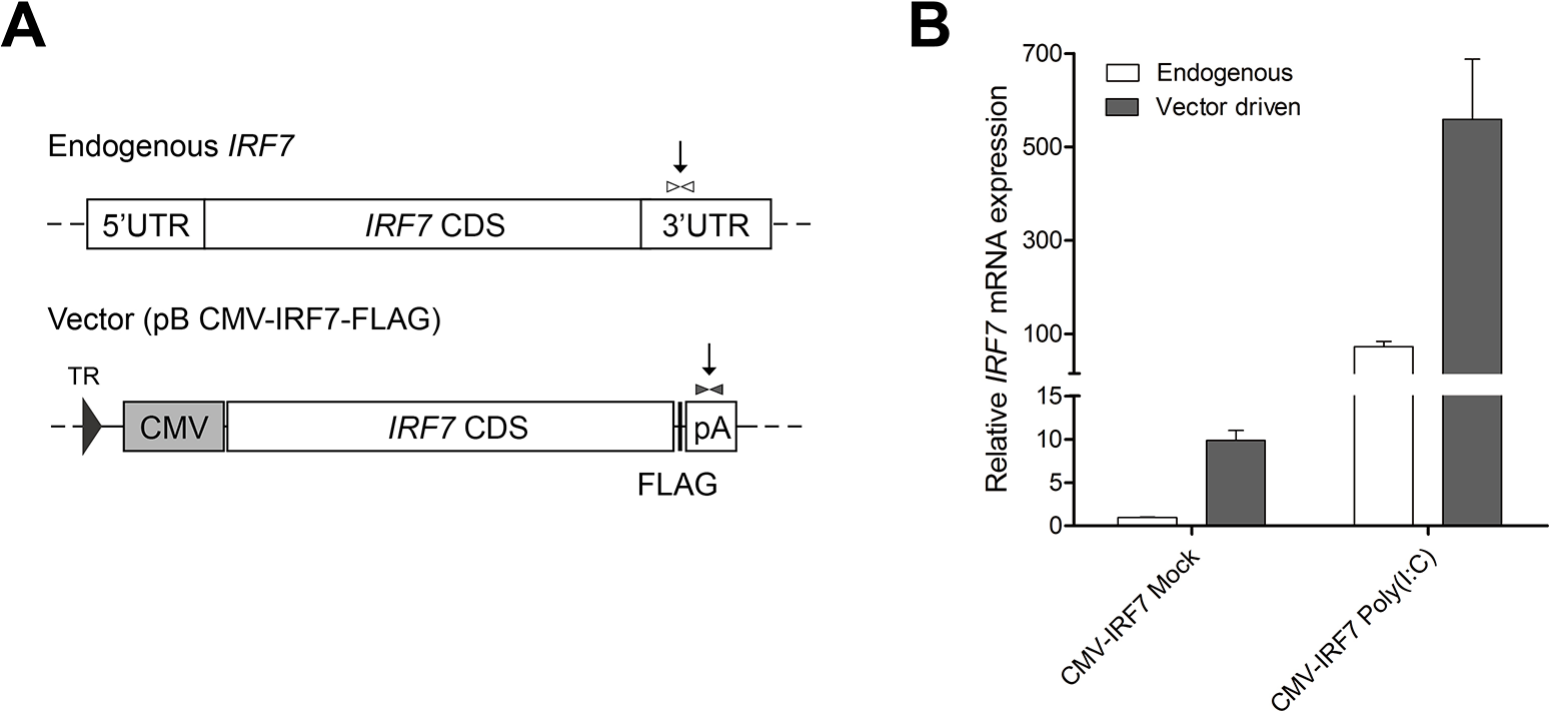
Characterization of IRF7 expression in the overexpression cell line. (A) Two different primer sets, specific to each endogenous or vector driven gene, were designed in the downstream region of the IRF7 CDS to differentiate between native versus construct driven expression. The locations of primers are indicated as arrow lines. (B) Relative quantitation differentiation of *IRF7* expression in the mock or poly(I:C) stimulated overexpression cell lines. Error bars indicate the SEM of triplicate analyses.

As can be seen in Fig 2A, poly(I:C) treatment induced expression of *IRF7* was up-regulated with similar magnitude in both the control and overexpression cell lines. In order to identify the origin of IRF7 expression in the poly(I:C) stimulated *IRF7* overexpression cell line, primers were designed to distinguish between endogenous promoter derived transcripts and vector CMV promoter derived transcripts (Fig 7A). In the absence of poly(I:C) stimulation, the ratio of endogenous to vector derived IRF7 was approximately 1:10; poly(I:C) stimulation resulted in an approximately 100 fold and 60 fold up-regulation of endogenous and vector-derived transcripts respectively (Fig 7B).

## Discussion

The overall goal of this study was to identify novel genes and signaling pathways potentially associated with pathogen induced, IRF7 modulation in chickens; such discoveries are important in laying a solid foundation for further understanding the molecular mechanisms of the antiviral response. In this study, an integrative high-throughput functional genomics approach was applied by combining overexpression and knockdown assays with RNA-seq; this side-by-side approach was taken in an effort to obtain corroboration and complementary support in the identification of potential genes that are under the control of IRF7.

Immortalized chicken embryonic fibroblast DF-1 cells were used to generate *in vitro* models for stable *IRF7* overexpression and knockdown studies. The DF-1 cell line has been widely used for viral infection studies because many avian viruses, such as AIV and NDV, can replicate in it [28, 29]. The expression of *TLR2*, *3*, *4*, and *7*, known triggers of IRF7 mediated antiviral pathways in various cell types, were detected in the DF-1 cell lines via RT-PCR (data not shown) [30]. Therefore, PGN, poly(I:C), LPS and Loxoribine which are the known ligands of each TLR were selected for stimulation of the cell lines to examine which pathways are associated with IRF7 modulation. Functional assays of *IRF7* expression and its effects were carried out at 6h post induction; this time-point was selected as it is in accordance with other work which has demonstrated that *IRF7* expression levels peak around 6-9h post infection in both high (H5N1) and low (H9N2) pathogenic AIV infected DF-1 cell lines [13].

The major function of IRF7 in mammals is regulating type I IFNs; however, little is known about the function of chicken IRF7 [30]. The *Irf7* ^-/-^ mouse model showed severe impairment of type I IFN expression in fibroblasts upon viral infections, suggesting that IRF7 is an important regulator of the IFN induction [7]. In this study, results from the overexpression and knockdown of IRF7 enabled us to propose that the chicken IRF7 also modulates type I IFNs despite the relatively low amino acid sequence similarity between chickens and mammals (38% similarity to humans). The expression of *IFNB* was significantly induced in cells overexpressing IRF7 upon poly(I:C) stimulation (Fig 4A), and knockdown of IRF7 resulted in significant down-regulation of *IFNA* under mock conditions (Fig 2D). Overexpression of IRF7 did not, however, result in the up-regulation of *IFNA* (Fig 2B), nor did the knockdown of IRF7 cause a change in the expression levels of IFNB (Fig 2D) in any of the conditions at 6h post induction. Our results may suggest that chicken IRF7 participates in mediating *IFNB* (but not *IFNA*) gene induction upon viral infection, and in controlling the basal level of *IFNA* (but not *IFNB*) gene expression in the fibroblast. In mammals, a canonical two-step positive-feedback type I IFN gene regulation model has been proposed, in which IRF7 is crucial for the initial induction of type I IFN genes, and the induced IFNs in turn secondarily activate IRF7 to ensure the full induction of type I IFNs [6]. Given the potential existence of such a positive feed-back loop in chicken, investigation of more time points post induction may provide additional insight to better understand the function of IRF7 and the machinery involved in type I IFN mediated antiviral pathways.

Our qRT-PCR results (Fig 2) show that *IRF7* expression was triggered by the dsRNA analog poly(I:C) in both the *IRF7* overexpression DF-1 cells and the control DF-1 cells; Loxoribine, a ssRNA analog, also appeared to be able to up-regulate *IRF7* expression to some extent, but only in the *IRF7* overexpression DF-1 cell line with a much smaller magnitude compared to that of poly(I:C). These results may suggest that the IRF7 mediated antiviral response is more responsive to dsRNA than ssRNA in the chicken fibroblasts; similar patterns have been observed for PRRs in the IFN mediated response of different cell types infected with AIV (ssRNA virus). Marcus et al., observed that only the dsRNA moiety from AIV, which could be present during either replication or primary transcription, induced IFN in primary chicken embryo cells, whereas the ssRNA genome itself did not induce IFN [31]. Up-regulation of the dsRNA sensing receptor TLR3 and type I IFNs has been observed in the brain, lung, and spleen tissues of H5N1 infected chickens [32]. Other *in vitro* studies, done in a variety of different cell types (e.g. leukocytes, splenocytes, DF-1, and the macrophage cell line HD11) further support this model of a dsRNA mediated, type I IFN response in chickens [32–35].

The number of DEGs observed due to overexpression of IRF7 was five times more abundant in response to poly(I:C) stimulation than to mock treatment (130 vs. 26, Fig 3A and 3C). This increase in the number of DEGs strongly indicates that the function or regulation of IRF7 is much more significant after PRR activation than under normal conditions; it also suggests that additional activation is required for chicken IRF7 to function in its full transcription activator capacity. In mammals, once PRRs recognize a viral infection, a signaling cascade leads to the phosphorylation of IRF7 in the cytosol and to the translocation of IRF7 homodimer or IRF3/7 heterodimer into the nucleolus where they activate the type I IFN pathway [6, 7, 36]. It is possible that modulation of chicken IRF7 involves a mechanism similar to that of mammals, including both phosphorylation and translocation; the possibility of IRF7 heterodimerization with other IRFs in chickens also warrants further study, as IRF3 is presumed to be absent [3, 11]. An additional possibility is that much like IRF1 does [5], chicken IRF7 alone could activate some genes without additional post-translational modification; this would be consistent with the existence of DEGs in *IRF7* overexpression cells relative to control cells in the absence of any ligand stimulation [37].

Two DEG lists (Control poly(I:C) vs. CMV-*IRF7* poly(I:C) and NS poly(I:C) vs. KD poly(I:C)) can be considered to contain the list of genes that are under the direct or indirect control of IRF7 upon stimulation by dsRNA, and can give insight into the function of IRF7 under virus infected conditions. By comparing the two DEG lists, we found evidence that at least seven genes are regulated by IRF7 in chickens. Of particular note, three genes (*OASL*, tetratricopeptide repeat and ankyrin repeat containing 1 (*TRANK1*), zinc finger and NFX1-type containing 1 (*ZNFX1*)) were present on both DEG lists, but with opposite directions of regulation between the overexpression line and knockdown line upon stimulation (S1 and S2 Tables). Results from qRT-PCR (Fig 6A–6D) confirmed that an additional 4 genes (*EIF2AK2*, *LY6E*, *MX1*, and *WIF1*) are also regulated in opposite directions in the stimulated overexpression line and knockdown lines. *MX1*, *OASL* and *EIF2AK2* are recognized for their role as ISGs; they are considered to be important interferon induced antiviral host immune response effectors which traps viruses, cleaves the viral RNA, and inhibits translation respectively [38]. *LY6E* is also a known ISG in mammals, and it has been implicated as a candidate resistance gene for Marek’s disease virus in chickens [39]. An *in vitro*, genome-wide RNAi study suggested a regulatory link between the WNT signaling pathway and the RLR mediated immune response; WIF1 has been assigned to the WNT protein modulator family [40]. Little is known about the function of either *TRANK1* or *ZNFX1* in chickens or in any other species; further investigation of these relatively novel genes could significantly impact our understanding of the innate antiviral immune response in general.

Several IRFs, including IRF1, IRF8, and IRF10, were observed on our list of DEGs (S1 Table); this is consistent with the fact that Type I IFN inducible IRFs are known to regulate the expression of IFN target genes [41]. IRF1 and IRF8 are well known ISGs that are important for the activation of a second group of ISGs in mammals; IRF10, however, is unique to chickens [5, 42, 43]. IRF10 is involved in the late stages of the immune response. It is thought that IRF10 regulates the transition from the innate to the adaptive immune response in chickens [43]; the IRF7 mediated modulation of IRF10 suggests that birds can utilize additional antiviral response mechanisms compared to those used by mammals.

Signal transducer and activator of transcription 1 (*STAT1*) was also in the list of DEGs controlled by IRF7. The canonical two-step positive-feedback type I IFN gene regulation model utilizes a Janus kinase (JAK) – STAT signaling cascade in mammals [6]. Once the type I IFN receptor (IFNAR) is activated by type I IFNs, the JAK-STAT signaling pathway is triggered, which in turn activates ISGs [6, 44]. In mammals, the activation of ISGs is achieved by the transcription factor IFN-stimulated gene factor 3 (ISGF3) complex, which is a heterotrimer of STAT1, STAT2 and IRF9 [42]. The JAK-STAT pathway is conserved in chickens [2], and up-regulation of STAT 1 by overexpressed IRF7 upon poly(I:C) stimulation could imply a conserved usage of the canonical pathway for type I IFN mediated ISG activation in chickens; however, IRF9, a component of transcription complex ISGF3, has not yet been annotated in chickens [3, 9]. A more complete genome assembly and annotation in chicken may one day help identify the missing IRF9 in birds. If IRF9 is not present in chickens, due to their reduced repertoire of immune genes [3], some other factor must exist that plays a role analogous to that of IRF9; discovery of chicken IRF9 or its analogous factor will help elucidate the precise mechanism of ISG stimulation in the chicken’s IRF7 mediated antiviral response.

To better understand the biological regulation of IRF7 in chickens, GO term enrichment analysis was conducted using the DEGs from all the comparisons (Fig 5). Besides the enrichment of several immune-related GO terms as expected, lipid metabolism related GO terms including Phospholipid Transport and Lipid Transport were also significantly enriched (Fig 5A). This result provides the first line of evidence of versatile functions of IRF7 in chickens, and is consistent with a similar observation in IRF7 knock-out mice [45].

The functional annotation of KEGG pathways enriched from the DEGs of poly(I:C) induced overexpression sets (Control poly(I:C) vs. CMV-*IRF7* poly(I:C)) contains two major innate pathogen-recognition receptors, RLR and TLR signaling pathways (Fig 5A). The induction of PRRs by IFNs results in pathogen sensing sensitization, this increased sensitivity is important for the IFN mediated antiviral response to fully take place across cells [46]. There are three different classes of PRRs (TLRs, RLR, and nucleotide-binding oligomerization domain (NOD)-like receptors (NLR)) that are important for sensing different PAMPs in the cells [27, 47], each of which was represented among the members of the DEG list (S1 Table).

TLRs are transmembrane proteins that play an important role in the recognition of viral pathogens. Each TLR exhibits distinct specificity to different PAMPs. In mammals, TLR3 is localized in endosomes, and upon recognizing viral dsRNA, it initiates a signal cascade that triggers type I IFN production via the activation of IRF3 and IRF7 [47]. Chicken TLR3 is a true orthologue of mammalian TLR3; comparative studies predict that its function and the signaling pathway it belongs to are likely conserved across multiple species [27, 48]. The magnitude of poly(I:C) induced TLR3 expression in our IRF7 overexpressed DF-1 line was 5 times greater than that of the control line (Fig 4). The up-regulation of TLR3 but no other TLRs in our DEG list (Control poly(I:C) vs. CMV-IRF7 poly(I:C)) could indicate that pathogen sensing sensitization feedback is controlled by IRF7 in chickens, and that chicken TLRs have a strong specificity to different PAMPs. This result is consistent with results from another study which demonstrated that *TLR3* mRNA expression is stimulated in DF-1 cell lines treated with type I IFNs, and that IFNA augments the sensitivity of DF-1 cells to poly(I:C) [32].

The RLR signaling pathway is another important cascade that regulates the type I IFN mediated antiviral response. RIG-I and interferon induced with helicase C domain 1 (IFIH1, also known as MDA5) are two important RLRs that mediate type I IFN induction in mammals by recognizing cytosolic viral RNAs [49–51]. In our study, we found that *IFIH1* was differentially expressed upon poly(I:C) stimulation in our overexpression cell line (CMV-*IRF7* Mock vs. CMV-*IRF7* Poly(I:C), Control Poly(I:C) vs. CMV-*IRF7* Poly(I:C)). Mammalian RIG-I responds to influenza virus whereas MDA5 serves as the sensor for AIV in chickens and activates IFNB in the context of missing RIG-I [34, 52]. Another RLR, DEXH box polypeptide 58 (*DHX58*, also known as LGP2), is a positive regulator of the MDA5-RNA interaction; it, along with LGP2 (involved in the recognition of AIV in chickens) was identified as a member of the DEG lists [34, 53, 54]. RLRs utilize the adaptor molecule mitochondrial antiviral signaling protein (MAVS), which signals through different virus activated kinases to activate the IRF3/7 mediated type I IFN antiviral response [47]. The ubiquitination mediated degradation of MAVS after stimulation of RLRs is important for the activation of IRF3/7; this ubiquitination of MAVS is carried out by the E3 ligase TRIM25 [55]. *TRIM25* was also identified in the DEG lists, and a recent study has shown that *TRIM25* is up-regulated in NDV infected chickens and fibroblasts [56]. Collectively, these results suggest TRIM25 is an important factor acting in the antiviral response in chickens. Since both TLR3 and MDA5 recognize dsRNA and help drive the type I IFN response, further studies to dissect the function of each receptor and downstream signaling pathway in different contexts may provide additional insight into the underlining mechanisms whereby the antiviral response is modulated in chickens.

Some NLRs function as intracellular PRRs while others function as regulators [57]. NLRC5 has been characterized as a regulator of the innate and adaptive immune response in mammals with diverse functions, including both the positive and negative regulation of the type I IFN response [58–60]. Some studies have indicated that chicken NLRC5 is involved in the antimicrobial immune response and that the knockdown of *NLRC5* results in the down-regulation of type I IFNs in macrophages [61, 62]. The up-regulation of *NLRC5* in this study could suggest a positive regulatory function for NLRC5 on IFNs; however, given that NLRC5 can also interact with RIG-1 and MDA5 in mammals to inhibit the RLR mediated type I IFN response [58], it is possible that the up-regulation of *NLRC5* may instead reflect an attempt to impede the overexpressed IRF7. Further study regarding the interaction between NLRC5 and MDA5 in chickens will help to clarify the function of NLRC5 in regulating the IRF7 mediated response.

In addition to *NLRC5*, three other negative regulators (N-myc and STAT interactor (*NMI*), TRAF-type zinc finger domain containing 1 (*TRAFD1*), and *OASL*) were found on both DEG lists using poly(I:C) stimulated overexpression cell lines (CMV-*IRF7* Mock vs. CMV-*IRF7* Poly(I:C), Control Poly(I:C) vs. CMV-*IRF7* Poly(I:C)). NMI promotes degradation of IRF7 via ubiquitination and negatively regulates excessive type I IFN induction [63], TRAFD1 is a known negative regulator of TLRs and the RLR signaling pathway [64], and mouse OASL1, a homolog of aforementioned antiviral effector ISG protein OASL, is known to inhibit translation of IRF7 and to negatively regulate the extreme production of type I IFNs [65]. The up-regulation of these negative regulators in our study suggests that a conserved negative feedback mechanism to modulate and prevent an excessive antiviral response in chickens may exist.

The number of DEGs observed in the *IRF7* knockdown dataset was considerably smaller than that observed for the *IRF7* overexpression dataset. Given that coverage of transcriptome improves significantly between 20M to 30M reads [66], the difference in the number of DEGs for each dataset may be a reflection of the smaller number of reads obtained for the knockdown dataset as compared to the overexpression dataset (15M vs. 28M). Redundancy of transcription factors that regulate the type I IFN response could be another reason for the smaller number of DEGs in the knockdown dataset [67]. A study using an IRF3, IRF5, IRF7, triple gene knockout mouse has revealed that the production of type I IFNs could be carried out in an IRF3/7 independent manner, and that IRF5 alone was sufficient to induce the type I IFN response [44]; this may partially explain why the effect of IRF7 knockdown on transcriptome changes was minimal. Alternatively, the incomplete knockdown of *IRF7*, means that the remaining 30% residual expression of IRF7 could have maintained a certain level of basal immune modulation. An ongoing IRF7 knock-out approach, applying CRISPR-Cas9, may be able to provide some insights on the different IRFs that are involved in controlling the type I IFN response in chickens.

In general, more biological replicates for differential expression analysis would increase the statistical power in the detection of DEGs [21]; however, the number of expected DEGs was small to begin with, as there was only a single gene modification in the nearly identical DF-1 cell population. Because this study was a first step in the candidate gene screening process for genes associated with IRF7, pooling of biological replicates was more deemed most efficient and cost-effective for our purposes.

It is known that a subset of ISGs can be induced directly by IRF3 or IRF7, independent of the IFNs; this redundancy ensures the induction of the antiviral response by counteracting the pathogens’ attempts to evade the host IFN response [46, 67, 68]. Additional studies are warranted, wherein chromatin immunoprecipitation (ChIP)-seq may be applied to more thoroughly identify the target genes and binding sites of IRF7, and to better understand how IRF7 regulates gene expression in the antiviral response in chickens.

## Conclusions

In summary, utilization of both stable overexpression and knockdown assays, combined with cutting-edge RNA-seq techniques, provides a powerful method to aid in the elucidation of the function of IRF7 in regulating the host response to pathogen infection in chickens. To the best of our knowledge, this is the first report using a genome-wide, comprehensive approach to identify candidate genes and pathways related to the function of IRF7 in chickens. The results revealed more than 130 strong candidate genes that may potentially be regulated by IRF7 and more than 120 genes that were up-regulated when IRF7 is overexpressed. Of these genes, more than half represent novel genes, in which this is the first time a potential link with IRF7 regulation has been reported. Our results demonstrate a link between IRF7, and regulation of the RIG-1-like receptor signaling pathway, the Toll-like receptor signaling pathway and the Apoptosis pathway in chickens; furthermore, the primary function of IRF7 as type I IFN regulator is demonstrated to be conserved in chickens. Our results also offer support for the existence of a conserved negative feedback mechanism for the regulation of the anti-virus response by IRF7 in chickens. Finally, in silico bioinformatic analysis of RNA-seq data from multiple sources further suggests that the *IRF3* gene may actually be missing in chickens and chicken IRF7 may have a distinct function compared to its mammalian orthologue. The precise mechanism of action of chicken IRF7 in the type I IFN mediated antiviral response needs to be further elucidated.

## Supporting Information

S1 Fig Differentially expressed (DE) genes. DEGs in the comparison of Control poly(I:C) vs. CMV-IRF7 poly(I:C) in the (A) RIG-1-like receptor (RLR) and (B) Toll-like receptor (TLR) pathways.
(TIF)

S1 Table Differentially expressed genes between comparisons in the overexpression data set. (XLSX)

S2 Table Differentially expressed genes between comparisons in the knockdown data set. (XLSX)
