# Correction: Functional Analysis of Chicken IRF7 in Response to dsRNA Analog Poly(I:C) by Integrating Overexpression and Knockdown

**DOI:** 10.1371/journal.pone.0137672

**Published:** 2015-09-02

**Authors:** Tae Hyun Kim, Huaijun Zhou

The images for Figs 5 and 6 are incorrectly switched. The image that appears as Fig 5 should be Fig 6, and the image that appears as Fig 6 should be Fig 5. The figure captions appear in the correct order.

Please see the corrected Fig 5 here.

**Fig 5:**
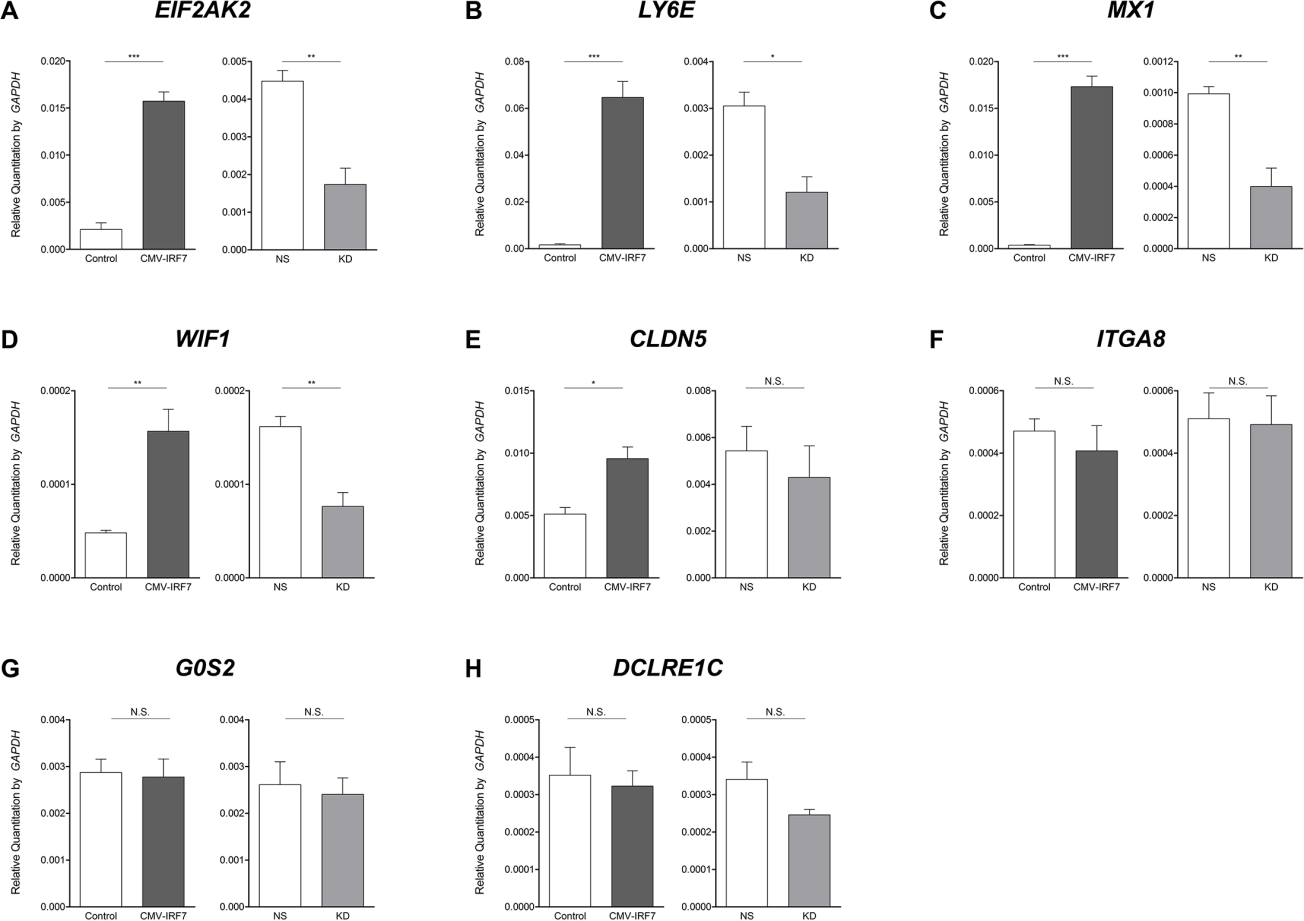
Validation of differentially expressed genes by quantitative reverse transcriptase PCR (qRT-PCR). Expression level of mRNAs were confirmed by qRT-PCR in the poly(I:C) induced overexpressed (left graph), knockdown (right graph) cell lines and their controls. (A) EIF2AK2; (B) LY6E; (C) MX1; (D) WIF1; (E) CLDN5; (F) ITGA8; (G) G0S2; (H) DCLRE1C. * P < 0.05, ** P < 0.01, *** P <0.001, N.S.: not significant. Error bars indicate the SEM of triplicate analyses.

Please see the corrected Fig 6 here.

**Fig 6:**
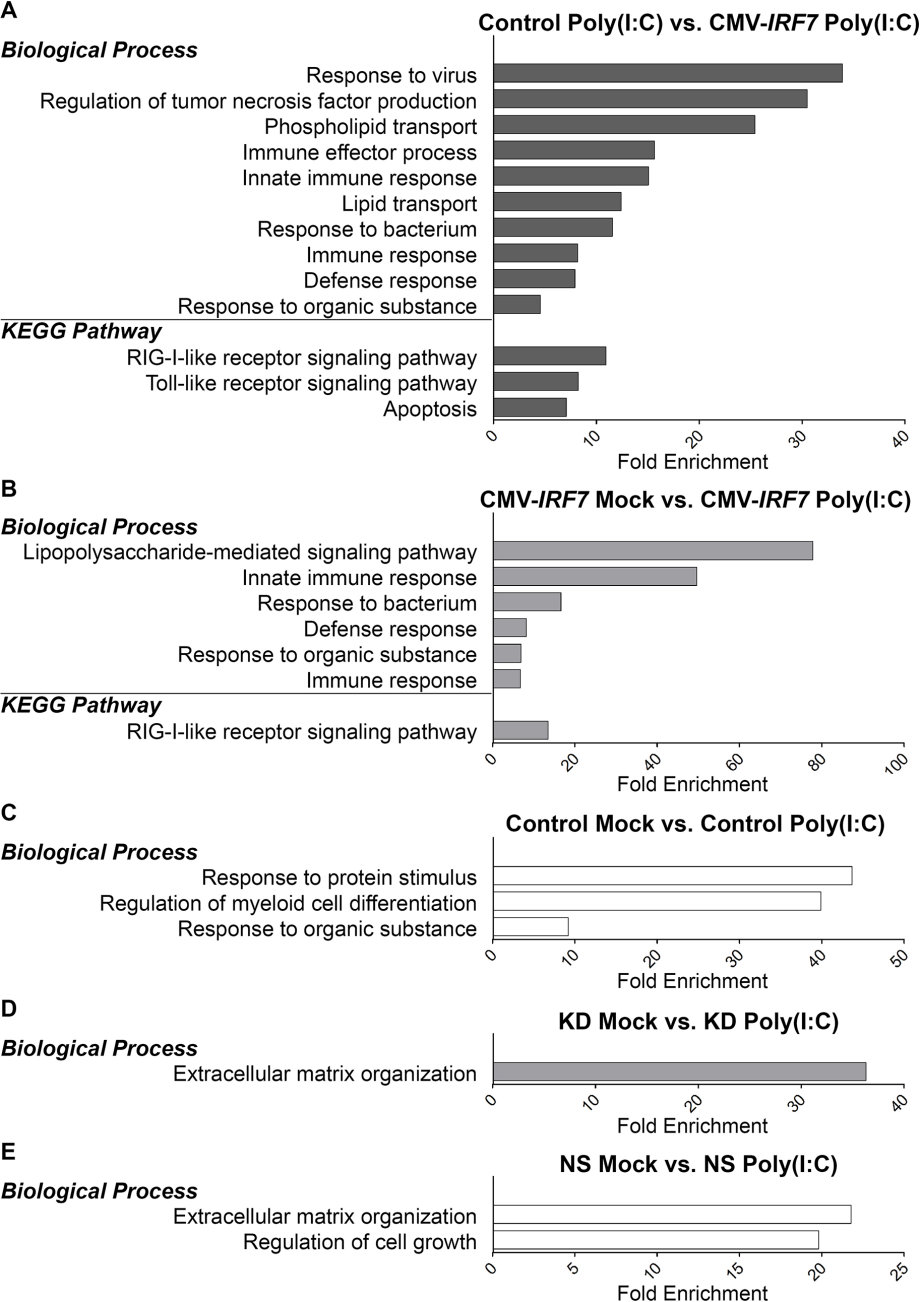
Gene ontology (GO) annotation terms on biological processes and pathways enriched by differentially expressed genes (DEGs). (A) GO terms enriched by DEGs between control and overexpressed cell lines upon poly(I:C) induction. (B) GO terms enriched by DEGs between mock and poly(I:C) treatments in the overexpressed cell lines. (C) GO terms enriched by DEGs between mock and poly(I:C) treatments in the control cell lines. (D) GO terms enriched by DEGs between mock and poly(I:C) treatments in the knockdown cell lines. (E) GO terms enriched by DEGs between mock and poly(I:C) treatments in the non-specific control cell lines. P<0.05.

There is an error in the fourth sentence of the sixth paragraph of the Discussion. The correct sentence is: Results from qRT-PCR (Fig 5A–5D) confirmed that an additional 4 genes (*EIF2AK2*, *LY6E*, *MX1*, and *WIF1*) are also regulated in opposite directions in the stimulated overexpression line and knockdown lines.

There are errors in the first and second sentence of the ninth paragraph of the Discussion. The correct sentences are: To better understand the biological regulation of IRF7 in chickens, GO term enrichment analysis was conducted using the DEGs from all the comparisons (Fig 6). Besides the enrichment of several immune-related GO terms as expected, lipid metabolism related GO terms including Phospholipid Transport and Lipid Transport were also significantly enriched (Fig 6A).

There is an error in the first sentence of the tenth paragraph of the Discussion. The correct sentence is: The functional annotation of KEGG pathways enriched from the DEGs of poly(I:C) induced overexpression sets (Control poly(I:C) vs. CMV-*IRF7* poly(I:C)) contains two major innate pathogen-recognition receptors, RLR and TLR signaling pathways (Fig 6A).
