# Supplementary figures and images for: Functional Analysis of Chicken IRF7 in Response to dsRNA Analog Poly(I:C) by Integrating Overexpression and Knockdown
Source: PLoS One. 2015 Jul 17;10(7):e0133450. doi: 10.1371/journal.pone.0133450 (PMC4505898; doi:10.1371/journal.pone.0133450)

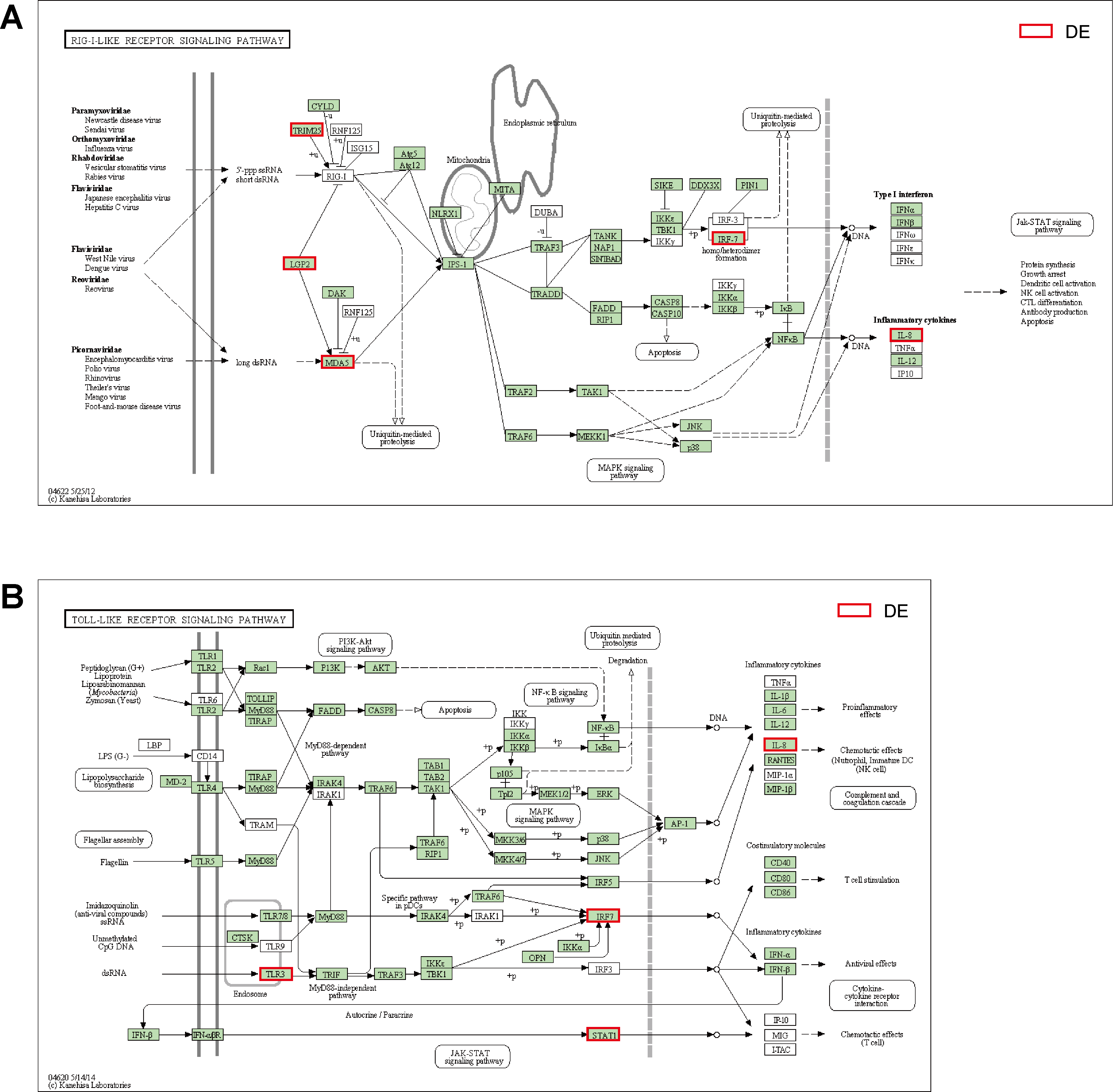

Supplement: S1 Fig — DEGs in the comparison of Control poly(I:C) vs. CMV-IRF7 poly(I:C) in the (A) RIG-1-like receptor (RLR) and (B) Toll-like receptor (TLR) pathways. (TIF) [file pone.0133450.s001.tif]
